# Crescentic Glomerulonephritis in Association With Renal Cell Carcinoma: A Case Report

**DOI:** 10.7759/cureus.67523

**Published:** 2024-08-22

**Authors:** Farah Sharieh, Hyun-Ryung Choi, Ezza Bashir, Hamza Bajwa, David Da Rocha, Saad Bajwa

**Affiliations:** 1 Family Medicine, Mercy Health St. Vincent Medical Center, Toledo, USA; 2 Nephrology, Mercy Health St. Vincent Medical Center, Toledo, USA; 3 Hospital Medicine, Mercy South Hospital, St. Louis, USA; 4 Nephrology, Renal Services of Toledo, Toledo, USA; 5 Internal Medicine, Rawalpindi Medical University, Rawalpindi, PAK

**Keywords:** focal necrotizing glomerulonephritis, renal cell carcinoma, smoking tobacco, acute lymphangitis, crescent shape glomerulonephritis

## Abstract

Clear renal cell carcinoma (RCC) is the most common primary renal tumor originating within the renal cortex. It is responsible for 75% to 85% of all primary renal neoplasms. Multiple risk factors are associated with the development of RCC, the most common being smoking. On some occasions, RCC has been linked to some autoimmune conditions, but data is limited. Especially, its association with glomerulonephritis (GN) is rare in literature and not fully understood. In this case report, we discuss a presentation of RCC associated with crescentic GN.

## Introduction

Renal cell carcinoma (RCC) could be primary or secondary. The most common primary renal cancer originates within the renal cortex and is responsible for 80% to 85% of all primary renal neoplasms. Clear cell carcinoma is the most common type of renal carcinoma, and it accounts for 75% to 85% of all renal tumors. Other cancers include papillary carcinoma (10-15%), chromophobe (5-10%), and oncocytoma (3-7%) [1,2].

RCC occurs predominantly in the sixth to eighth decade of life and is twice more common in males than females. There are multiple reported factors associated with a higher risk of developing RCC. These include, but are not limited to, smoking, obesity, hypertension, chronic kidney disease or acquired cystic diseases, analgesics, occupational exposure to compounds such as asbestos, and genetic or hereditary factors. On rare occasions, RCC has been linked to some autoimmune conditions, but data is limited [3].

In this case report, we discuss a rare presentation of RCC associated with crescentic glomerulonephritis (GN).

## Case presentation

Our patient is a 46-year-old Caucasian male who noticed a marble-sized swelling on his penis after waking up one morning. Physical examination demonstrated focal swelling of about 2x3 cm on the dorsal surface of the penile shaft near the base without discharge or evidence of lesions or ulceration. The initial laboratory studies and infectious workup were negative, but urinalysis was positive for blood and 3+ proteinuria. His personal history was significant for 20 pack-year smoking. He worked as a biologist in a laboratory where his job included handling various animals including dogs, cats, guinea pigs, hamsters, rabbits, and bats. He was advised to undergo imaging studies and blood tests but he did not follow up.

**Figure 1:**
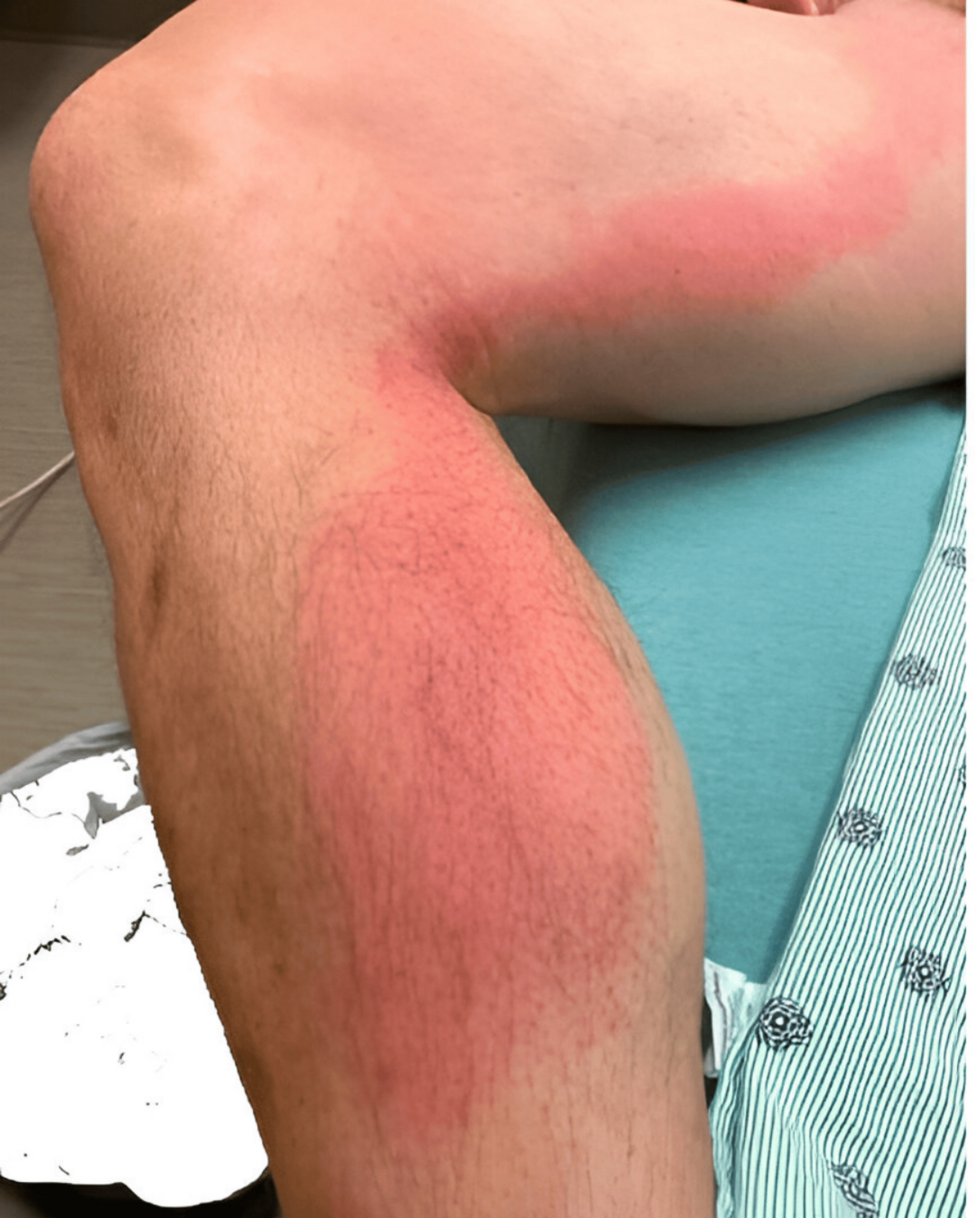
Ascending erythema of right lower extremity

Two months after his initial presentation, he woke up with a 3-4 cm area of redness on his right medial calf. Within a couple of days, the erythema progressed in a linear fashion upward along his right medial thigh up to the inguinal crease as shown in Figure 1.

This was associated with gross hematuria. He denied recent travel, acute febrile illness or exposures, bug or tick bites, or scratches from animals. The urinalysis again showed a large amount of blood, 4+ protein, trace leukocyte esterase, and a white blood cell count of 20-50/HPF (high power field). The physical examination was significant for non-tender linear erythema extending along the right medial leg and thigh up to the inguinal crease. He also had palpable right inguinal lymph nodes but no flank pain or palpable abdominal mass. No scrotal edema, swelling, or varicocele was noted. Venous Dopplers of bilateral lower extremities were negative for deep venous thrombosis. The leg erythema was diagnosed as acute lymphangitis; he was admitted to the hospital and was started on intravenous antibiotics. His laboratory work-up is shown in Tables 1-3.

**Table 1:** Initial laboratory work-up BUN: blood urea nitrogen; CRP: C-reactive protein

Laboratory tests	Reference range	Result
BUN	6-20 mg/dL	12
Creatinine	0.70-1.20 mg/dL	1.16
Hemoglobin	13.0-17.0 g/dL	18
White blood cells	3.5-11.3 k/uL	9.1
Platelets	138-453 k/uL	161
Erythrocyte sedimentation rate	0-15 mm/hr	25
CRP	0.0-5.0 mg/L	47.7

**Table 2:** Urinalysis with microscopy (pertinent results only) UA: urinalysis; RBCs: red blood cells; WBCs: white blood cells; HPF: high power field; LPF: low power field

Component	Reference range	Results
Color, UA	Yellow	Orange
Urine, Hgb	Negative	Large
Urine, Protein	Negative	4+
Leukocyte esterase	Negative	Trace
WBCs	0-5/HPF	20-50/HPF
RBCs	0-2/HPF	Too numerous to count
Casts	0-2/LPF	20-50 (Hyaline)

**Table 3:** Autoimmune workup ANA: antinuclear antibodies; ENA: extractable nuclear antigen; ds DNA: double-stranded deoxyribonucleic acid; ANCA: antineutrophilic cytoplasmic antibody; U1RNP: U1 small nuclear ribonucleoprotein; IU: international unit; mg: milligram; mL: milliliter; AU: arbitrary unit

Serologic markers	Reference range	Result
ANA	Negative	Negative
ENA antibodies screen	<0.7 U/mL	0.2
Anti-dsDNA	<10.0 IU/mL	<0.5
Complement C4	10-40 mg/dL	23
Complement C3	90-180 mg/dL	126
ANCA myeloperoxidase	0.0-3.5 AU/mL	1.2
ANCA proteinase 3	0.0-2.0 AU/mL	<0.7
Anti-U1RNP	<5.0 U/mL	0.8

**Figure 2:**
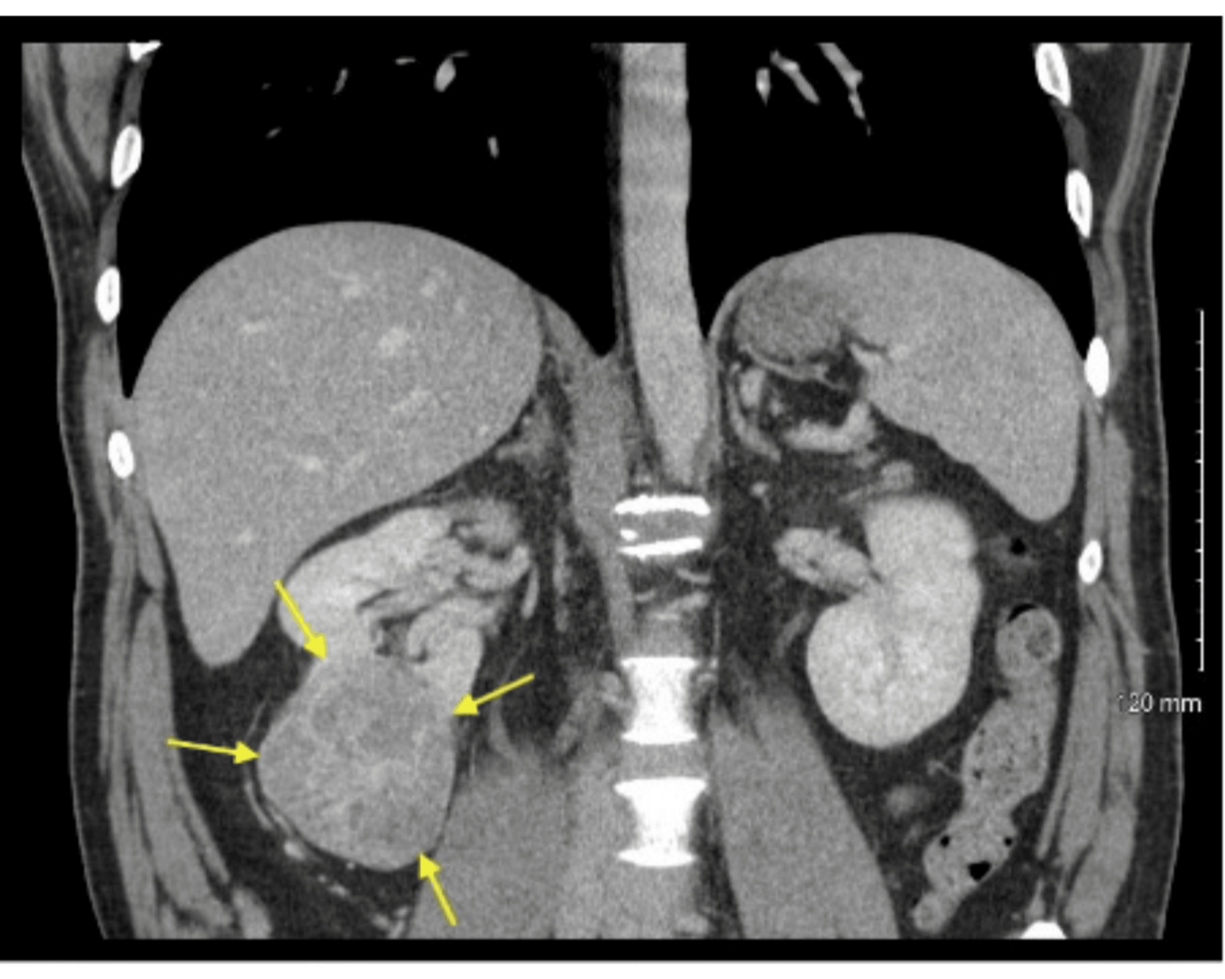
CT abdomen pelvis shows a solid mass attached to the lower pole of the right kidney (yellow arrows).

Renal ultrasound showed a solid mass attached to the lower pole of the right kidney measuring up to 8.6 cm in the greatest dimension. CT abdomen and pelvis with intravenous contrast showed a 6.9 x 7.2 x 6.0 cm heterogeneous enhancing mass along the inferior pole of the right kidney as shown in Figure 2. No bladder mass was found on the CT urogram. The preliminary diagnosis of renal carcinoma was made.

During the patient’s hospital course, his lower extremity erythema was significantly improved after three days of intravenous antibiotics. He was followed up closely by urology and underwent elective right radical nephrectomy. The pathology from the resected kidney confirmed grade 3 renal clear cell carcinoma. The report also mentioned that “the non-neoplastic portion of the kidney had destructive GN with both active cellular crescents and more remote fibrous crescents. The involved glomeruli had mild neutrophilic or mononuclear glomerular inflammation. Paraffin immunofluorescence (IF) showed non-dominant mesangial IgA staining, but no definitive electron-dense deposits on reprocessed tissue.” He was followed up closely and a repeat CT abdomen four months after right nephrectomy showed no evidence of recurrence or local metastatic disease.

## Discussion

This case highlights several important associations in patients with RCC. There are multiple important risk factors for RCC development, of which smoking is not only associated with increased risk of developing RCC, but also the number of daily cigarettes consumed is directly associated with advanced disease at presentation [4].

The classic presentation of RCC includes gross hematuria, vague abdominal pain or dragging sensation, and/or weight loss. A palpable abdominal mass is a less common presentation. However, many patients remain asymptomatic until late in the disease progression with or without evidence of distant metastasis. Urinalysis typically shows microscopic hematuria and variable proteinuria. Imaging with a CT abdomen and pelvis is the initial diagnostic modality combined with a CT urogram to rule out bladder mass or involvement. Definite diagnosis is made on kidney biopsy [5].

Our patient had a significant smoking history but no diagnosed structural or functional renal disease before this presentation. It is fair to assume that the GN in his right kidney was present for a while; however, the extent to which it may have influenced the oncologic pathway remains unclear.

**Figure 3:**
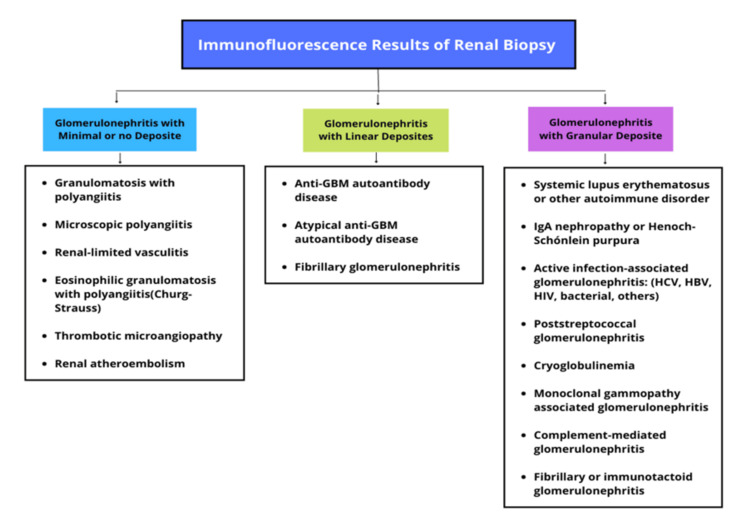
Immunofluorescence patterns of different types of immune complex deposition GBM: glomerular basement membrane; HCV: hepatitis C virus; HBV: hepatitis B virus; HIV: human immunodeficiency virus This figure is the original work of the authors.

The biopsy finding of crescentic GN refers to the extracapillary proliferation of more than 50% of glomeruli. It tends to rapidly progress to end-stage renal disease. There are three types of crescentic GN based on IF results as explained in Figure 3. The mechanism of injury is different in all three types; however, they all lead to the crescent formation and the similar renal manifestation of rapidly progressing GN [6-8].

The differential diagnosis in the context of this renal biopsy results would include antineutrophilic cytoplasmic antibody (ANCA)-related GN and IgA nephropathy. Chronic infection-related GN and other immune deposition diseases were less likely. In postinfectious GN, electron microscopy (EM) would show subepithelial humps and positive antistreptococcal antibodies which were not apparent in this case. Moreover, in lupus nephritis immune deposits are usually "full house" with IF, and EM demonstrates mesangial and subendothelial immune complex deposits. In mixed cryoglobulinemia, there is evidence of circulating cryoglobulins and intraluminal "thrombi" within glomerular capillaries [2]. IgA nephropathy represents the most common glomerulopathy worldwide and is responsible for approximately 10% of GN in the United States [9]. IgA nephropathy typically presents with more extensive mesangial expansion and hypercellularity at a relatively younger age, as well as more extensive IgA staining by IF. The lack of extensive mesangial deposition with IgA on IF precludes IgA nephropathy and made this diagnosis less likely in our patient.

The biopsy results also showed temporal heterogeneity and relatively mild glomerulitis with pauci-immune deposits on IF and EM, which usually favors an ANCA-related GN. ANCA-associated small vessel autoimmune conditions include granulomatosis with polyangiitis (GPA), previously known as Wegener's granulomatosis, microscopic polyangiitis, and eosinophilic GPA (formally known as Churg-Strauss syndrome). Furthermore, ANCA vasculitis can be classified into proteinase 3 (PR3-ANCA) and myeloperoxidase (MPO-ANCA) which are cytoplasmic antigens that are present at the surface of cytokine-stimulated leucocytes.

Several associations of renal cancers with vasculitis have been reported. RCC seems particularly strongly associated with GPA [10,11]. This association remains unclear but possible postulated mechanisms include underlying undiagnosed tumors whose antigens may induce vasculitis. Additionally, potential side effects of immunosuppressive drugs such as cyclophosphamide therapy may initiate or energize tumor cells [10,11]. Our patient was not on any immunosuppressive therapy at the time of the diagnosis of RCC.

Autoimmune workup revealed that our patient had ANCA-negative, pauci-immune crescentic GN limited to the kidney (renal-limited vasculitis) which accounts for approximately 10% of patients with GN. One study found that ANCA-negative pauci-immune crescentic GN may be due to abnormalities of the alternative pathway of complement [11]. While complement activation is noted in ANCA-associated vasculitis, it is hypothesized that complement activation may be more prominent in ANCA-negative GN. The larger amount of C3 and a moderate amount of C9 in ANCA-negative GN implies activation of the alternate and terminal pathways of the complement system, suggesting that this entity may be caused or promoted by a genetic or acquired defect in the alternative pathway [11]. The generation of C5a, produced by the final complement pathway, plays a pivotal role in ANCA-mediated lesions leading to neutrophil activation and tissue damage. Although C3 levels were not decreased in our patient, the literature shows C3c is only deposited in approximately one-third of ANCA-associated vasculitis patients. The histologic findings and prognosis in ANCA-negative renal vasculitis are comparable with those of ANCA-positive disease [12].

Another term "idiopathic" rapidly progressive GN is implied in two settings: an immune complex disease that does not fit into any of the identifiable categories and a pauci-immune disease that is ANCA negative. The former is rare, while the latter accounts for less than 5% of cases of crescentic GN [13].

As mentioned earlier, the initial presentation of this patient at the hospital was for acute lymphangitis. However, RCC has been associated with lymph node involvement in advanced disease. In this case, the patient had a clear cell RCC grade 3 with no signs of metastasis on imaging and biopsy, and it is unlikely that the patient developed a paraneoplastic phenomenon which makes us believe that no correlation exists between the two conditions. We believe his lymphangitis was likely due to a biological exposure related to his occupation. Furthermore, infectious etiologies including HIV and hepatitis infections were also ruled out. The patient did not have recent toxin exposure, nor was he started on any new medications.

## Conclusions

The renal biopsy findings of necrotizing crescentic GN may have coexisted or preceded renal carcinoma. The causative relation between pauci-immune ANCA-negative GN and RCC is not fully understood. Our case presentation underscores the importance of precise diagnosis and the utility of renal biopsy in the diagnoses of renal vasculitis with high clinical suspicion in a set of patients who are ANCA negative. There should be a heightened awareness of the association between ANCA vasculitis and renal carcinoma. Further studies are required to explore this association.
